# Supplementary material for: Olfactory marker protein directly buffers cAMP to avoid depolarization-induced silencing of olfactory receptor neurons
Source: Nat Commun. 2020 May 4;11:2188. doi: 10.1038/s41467-020-15917-2 (PMC7198493; doi:10.1038/s41467-020-15917-2)
Supplement: Supplementary file 1 — Supplementary Information [file 41467_2020_15917_MOESM1_ESM.pdf]

Olfactory marker protein directly buffers cAMP to avoid depolarization-induced silencing of olfactory receptor neurons

Noriyuki Nakashima et al.

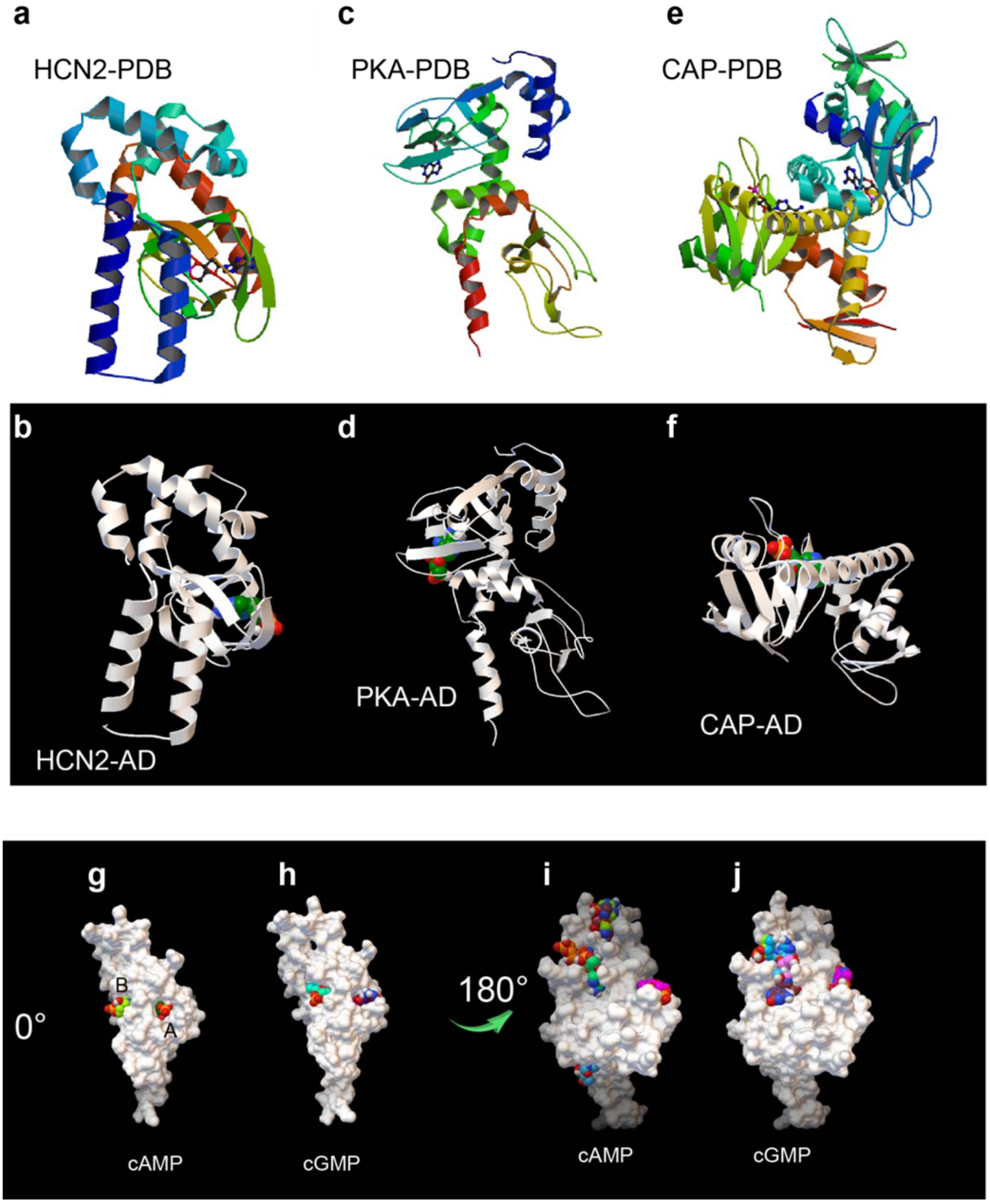

a
HCN2-PDB
c
PKA-PDB
e
CAP-PDB
b
HCN2-AD
d
PKA-AD
f
CAP-AD
g
h
i
j
0°
B
A
180°
cAMP
cGMP
cAMP
cGMP

Supplementary Figure 1

Validation of the simulation software. a-f, Experimentally determined conformations based on Protein Data Bank data (PDB**; a**, **c**, and **e**) and predicted conformations based on an AutoDock simulation (AD; **b**, **d**, and **f**) of a partial domain of HCN2 (**a**, **b**), a regulatory subunit of PKA (C and D) and CAP (**e**, **f**) in the presence of cAMP. In (**b**), (**d**) and (**f**), the proteins are shown with white ribbons and cAMP is shown using a coloured space-filling model. CAP is shown as a dimer in (**e**) and a monomer in (**f**). Panel (**a)** is the same as that in Fig. 1a. **g-j**, Simulated conformations of OMP with cAMP and another representative second cyclic nucleotide, i.e., cGMP, on the front (**g, h**) and back (**i, j**) sides of OMP. The simulated ΔG values of cGMP are -29.7 and -24.6 kJ/mol.

a

Gene Accession	Direction	Primer sequences (5' to 3) ATG : START	STOP	Restriction
OMP-Wt	Forward	GCTCGAGCCGCCACCATGGCAGAGGAT		XhoI
(NM_011010)	Reverse	CGTCGACGGTCAGAGCTGGTTAAAC	STOP	SalI
ΔOMP	Forward	GCTCGAGCCTGGGCCATGGCAGAGGATGG		XhoI
	Reverse	GGCCGCCGAGGCCGAGCTGGTTAAACACCACAG	deleted	SfiI
OMP-Mut	Forward	GAGTTTGAGGAGCGCCTTTC		
G126E	Reverse	GAAAGGCGCTCCTCAAACTC		
OMP-Mut	Forward	GGCCAAGATCGAAAAGGTCATG		
R136E	Reverse	CATGACCTTTTCGATCTTGGCC		
OMP-Mut	Forward	AACTGGACGCCCAAGCTCAC		
D90K	Reverse	ATGAGGTTGGTGAGCTTGGG		
CNGA2	Forward	GAATTCGCCACCATGATGACCGAAAAATCCAACGGTGTG		EcoRI
(NM_007724)	Reverse	GTCGACCTATTCAGCAACAGCTGGCTCAG	STOP	SalI
Rluc	Forward	GAATTCTTGCCACCATGGCTTCCAAGGT		EcoRI
(pGL4.74)	Reverse	GCGGCCGCTTACTGCTCGTTCTTCAGCA	STOP	NotI
Δ Rluc	Forward	CTCGAGTGGTGGTGCCACCATGGCTTCCAAGGT		XhoI
	Reverse	GTCGACCACCACCCTGCTCGTTCTTCAGCA	deleted	SalI

b

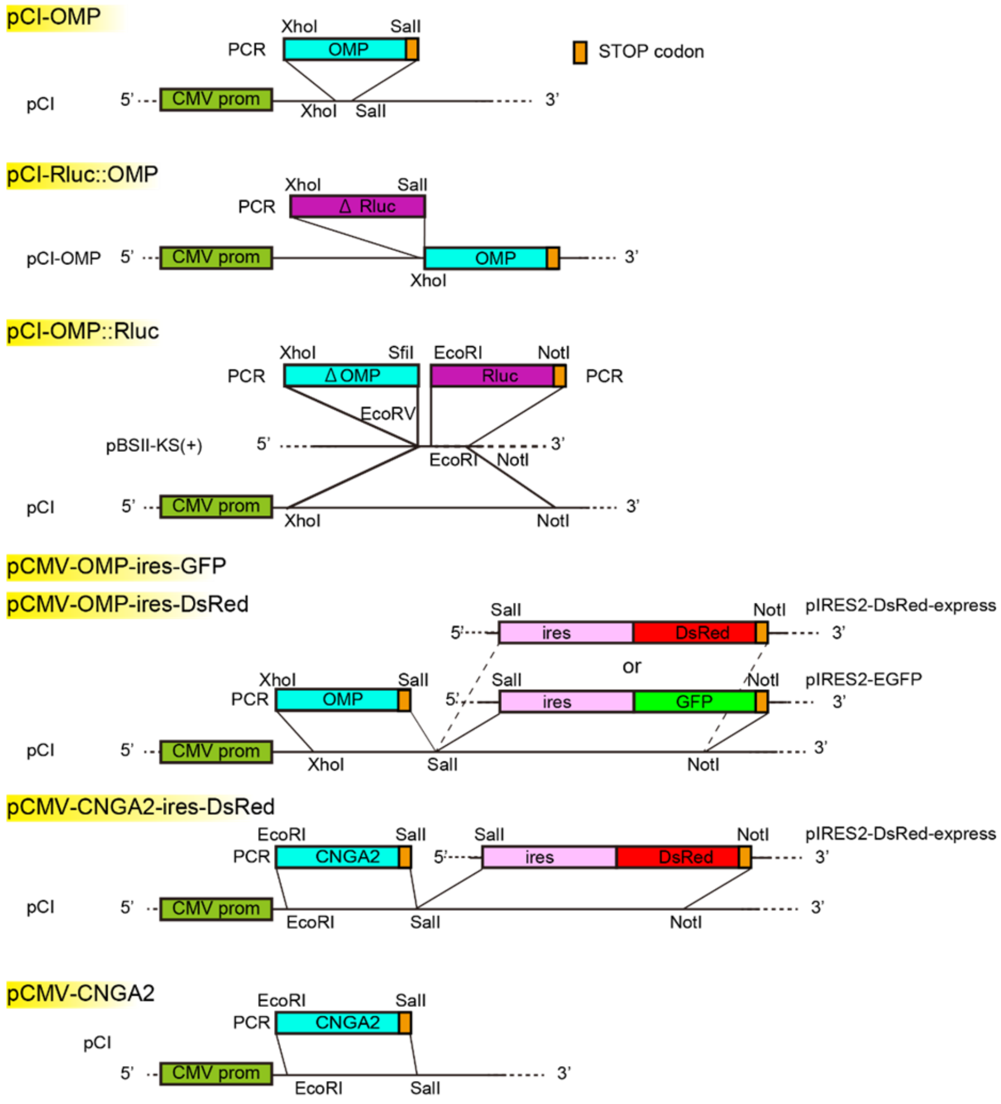

Supplementary Figure 2

Schematic of the methods used for construction of the vectors used in this study.

a, Primers used for cloning the cDNAs of OMP and CNG2A and subcloning three mutated OMP cDNAs (OMP-mt), including STOP codon-deleted Rluc (ΔRluc) fused with OMP and STOP codon-deleted OMP fused with Rluc. The primers used in the present study are listed in the order of their appearance in the manuscript. 3’ UTR, downstream untranslated region. The coloured bases correspond to the description in the same colour. **b**, Graphical procedures used for vector construction. CMV prom, CMV promoter; UTR, untranslated region; Rluc::OMP and OMP::Rluc, cDNAs in the 5’ to 3’ direction corresponding to the fused proteins (Rluc-OMP and OMP-Rluc, respectively) in the N to C direction.

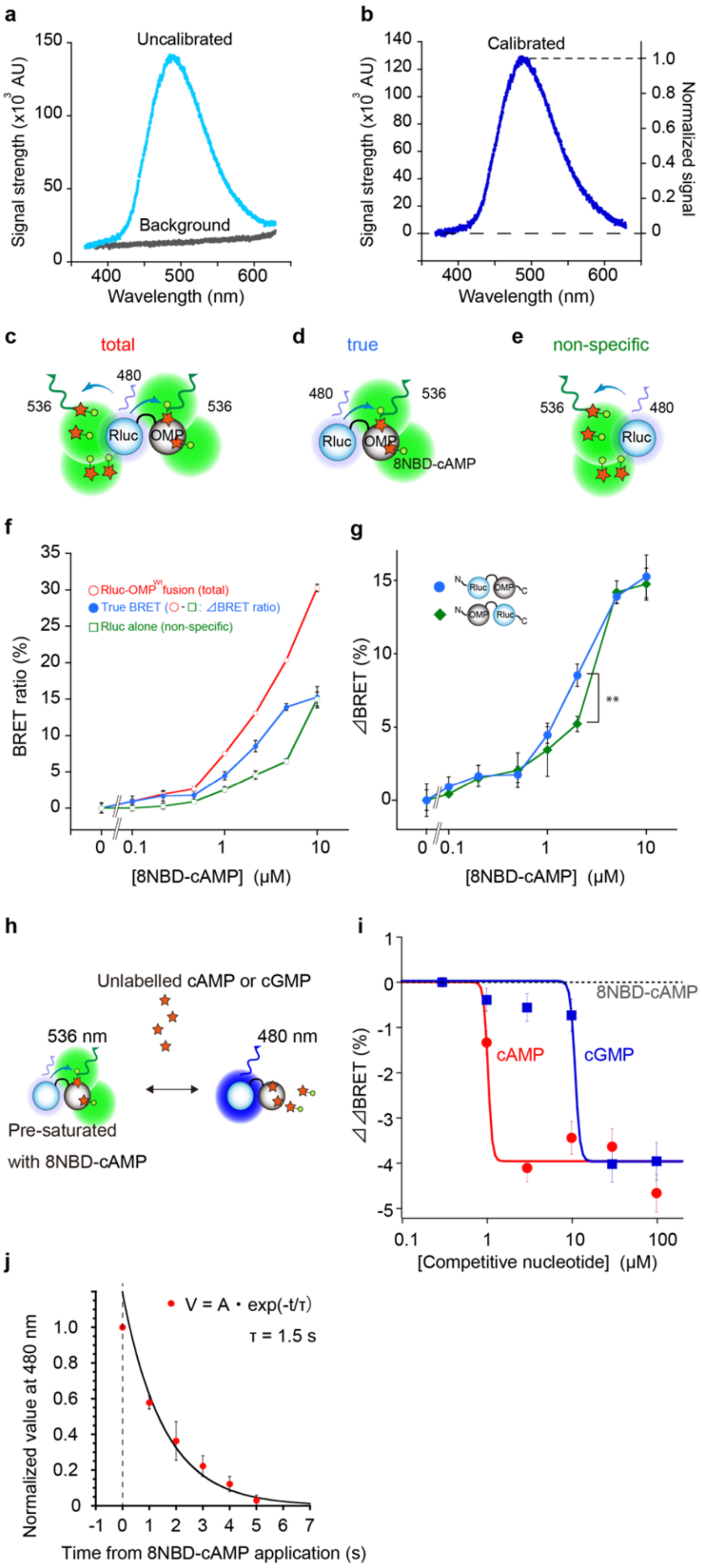
a
Uncalibrated
Background
Signal strength (x10³ AU)
Wavelength (nm)
b
Calibrated
Signal strength (x10³ AU)
Normalized signal
Wavelength (nm)
c
total
d
true
e
non-specific
Rluc
OMP
8NBD-cAMP
f
Rluc-OMP^wt fusion (total)
True BRET (○-□: ⊿BRET ratio)
Rluc alone (non-specific)
BRET ratio (%)
[8NBD-cAMP] (μM)
g
⊿BRET (%)
[8NBD-cAMP] (μM)
h
Unlabelled cAMP or cGMP
536 nm
480 nm
Pre-saturated
with 8NBD-cAMP
i
8NBD-cAMP
cAMP
cGMP
⊿⊿BRET (%)
[Competitive nucleotide] (μM)
j
V = A・exp(-t/τ)
τ = 1.5 s
Normalized value at 480 nm
Time from 8NBD-cAMP application (s)

Supplementary Figure 3

Procedures used to analyse the BRET signals.

a, Representative luminescence emission spectra of Rluc-OMP with (uncalibrated) and without (background) a substrate. **b**, Representative calibrated emission spectrum of Rluc-OMP determined through background subtraction. **c-e**, Schemes of (**c**) total, (**d**) true and (**e**) nonspecific BRET signals. **f**, Dose responses of total (n = 8, 6, 25, 26, 23, 24 and 24 plates for 100 nM, 200 nM, 500 nM, 1 µM , 2 µM, 5 µM, and 10 µM, respectively), nonspecific (n = 11, 10, 10, 10, 13, 17, 9 and 9 plates for 0, 100 nM, 200 nM, 500 nM, 1 µM , 2 µM, 5 µM, and 10 µM, respectively) and true (subtraction) BRET ratios relative to the level without 8NBD-cAMP. True BRET signals (ΔBRET) were obtained by subtracting the nonspecific signals from the total signals. **g**, OMP with Rluc fused to the C-terminus (OMP-Rluc; green-filled diamonds, n = 3) also showed BRET responses similar to those of Rluc-OMP (n = 23, 26, 18, 9, 26, 26 and 8 plates for 100 nM, 200 nM, 500 nM, 1 µM , 2 µM, 5 µM, and 10 µM, respectively). Two-sided T-test; P = 0.443, 0.227, 0.209, 0.246, 0.456, 0.005, 0.440 and 0.458 from 0 to 10 µM, respectively. **h,** Competitive assay schemes. Unlabelled cAMP or cGMP competes with 8NBD-cAMP, resulting in a decrease in BRET signals (ΔBRET ratio). **i,** The ΔΔBRET ratio of cAMP (same as Fig. 1 h) or cGMP as a competitor was fitted by single sigmoidal curves. Two-sided unpaired T-test; **P = 0.0047; n = 3 each. **j**, Time course of the normalized decrease in the 480-nm spectral signals upon application of 8NBD-cAMP (µM). The spectrum of limited wavelengths was measured at 1-s intervals. The values were fitted with a single exponential curve, yielding a time constant of 1.5 s. (n = 4 independent plates). Mean ± s.e.m.

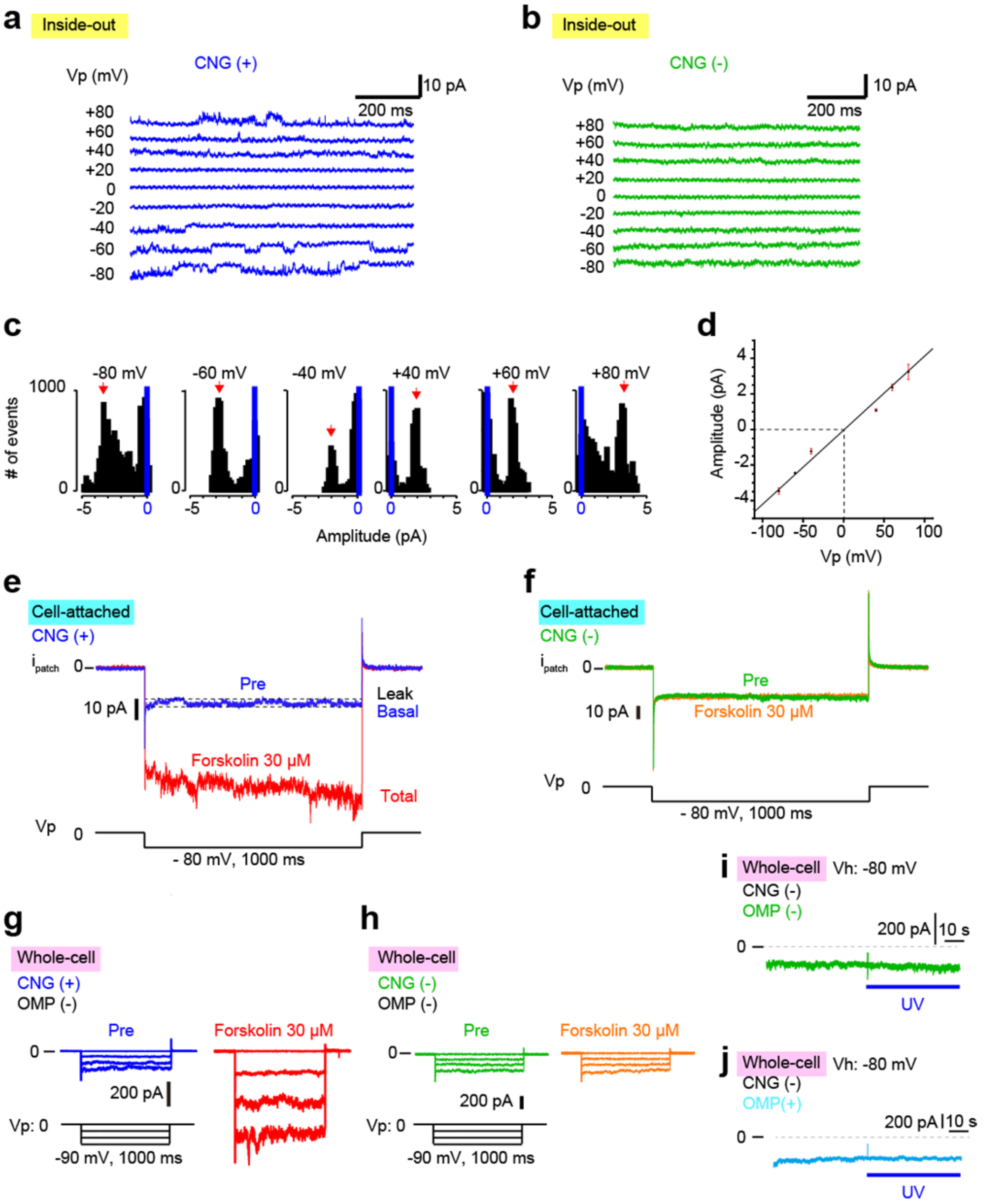
a
Inside-out
CNG (+)
Vp (mV)
10 pA
200 ms
+80
+60
+40
+20
0
-20
-40
-60
-80
b
Inside-out
CNG (-)
Vp (mV)
10 pA
200 ms
+80
+60
+40
+20
0
-20
-40
-60
-80
c
# of events
1000
-80 mV
-60 mV
-40 mV
+40 mV
+60 mV
+80 mV
Amplitude (pA)
d
Amplitude (pA)
Vp (mV)
e
Cell-attached
CNG (+)
i_{patch}
10 pA
Pre
Leak
Basal
Forskolin 30 μM
Total
Vp
- 80 mV, 1000 ms
f
Cell-attached
CNG (-)
i_{patch}
Pre
10 pA
Forskolin 30 μM
Vp
- 80 mV, 1000 ms
g
Whole-cell
CNG (+)
OMP (-)
Pre
Forskolin 30 μM
200 pA
Vp: 0
-90 mV, 1000 ms
h
Whole-cell
CNG (-)
OMP (-)
Pre
Forskolin 30 μM
200 pA
Vp: 0
-90 mV, 1000 ms
i
Whole-cell
Vh: -80 mV
CNG (-)
OMP (-)
200 pA
10 s
UV
j
Whole-cell
Vh: -80 mV
CNG (-)
OMP(+)
200 pA
10 s
UV

Supplementary Figure 4

Successful transfection of CNGA2 cDNA into HEK293T cells.

a, **b**, Representative traces of excised inside-out voltage clamp recording within a bath solution containing an external solution using HEK293T cells transfected with (**a**) or without (**b**) CNGA2 channel cDNA. Patch membranes were held at different holding pipette potentials (Vp). Stepwise channel activities were observed only in the cells transfected with CNGA2 channel cDNA (**a**). **c**, Histograms of current amplitudes obtained from experiments in (**a**). Events corresponding to stepwise activities were counted, and the total sample numbers differed at different holding potentials. **d**, The current steps corresponding to a single-channel activity level were averaged and plotted against the holding potential (Vp). A linear approximation yielded a slope of 41.9 pS. n = 5 patches. Mean ± s.e.m. **e**, **f**, Cell-attached voltage clamp recording from the patch membrane containing multiple channels. In the cells with CNGA2 cDNA, basal channel activities were observed, which were further sensitized by the application of an AC activator (30 μM forskolin). Cells without cDNA showed no basal channel activities or forskolin-sensitive currents. **g**, Whole-cell voltage clamp recording from cells in which CNG2A cDNA was transfected. Forskolin induced an obvious current increase. **h,** Whole-cell voltage clamp recording from the cells without transfected cDNAs. Forskolin induced no obvious current increase. **i**, Whole-cell recording from the cells with neither cDNAs for OMP nor CNG showed no response to UV flash in the presence of caged cAMP in the recording pipette. **j**, Whole-cell recording from the cells with OMP cDNA but without CNG cDNA showed no response to UV flash in the presence of caged cAMP in the recording pipette. Command pulses are shown below the representative current traces in (**e**-**f**).

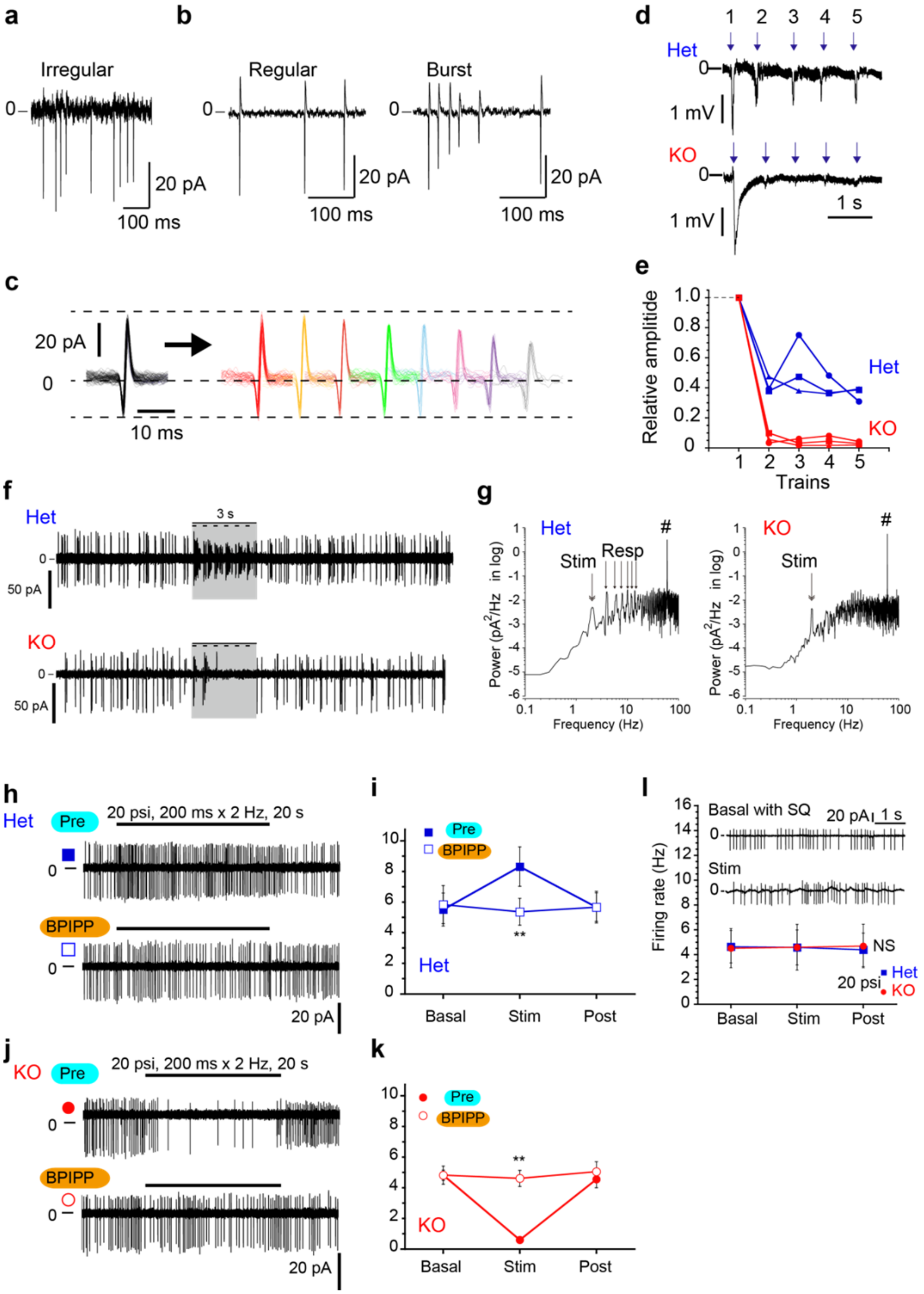

a
Irregular
20 pA
100 ms
b
Regular
Burst
20 pA
100 ms
d
1 2 3 4 5
Het
1 mV
KO
1 s
c
20 pA
10 ms
e
Relative amplitude
Het
KO
Trains
f
Het
3 s
50 pA
KO
g
Het
Stim
Resp
#
KO
Power (pA2/Hz in log)
Frequency (Hz)
h
Het
Pre
20 psi, 200 ms x 2 Hz, 20 s
BPIPP
20 pA
i
Pre
BPIPP
Het
Basal
Stim
Post
l
Basal with SQ
20 pA
1 s
Stim
Firing rate (Hz)
NS
20 psi
Het
KO
j
KO
Pre
20 psi, 200 ms x 2 Hz, 20 s
BPIPP
20 pA
k
Pre
BPIPP
KO
Basal
Stim
Post

Supplementary Figure 5

Responses of ORNs in Het and KO mice.

a, Representative traces of irregular firings with fluctuating spike amplitudes. **b**, Representative trace of regular firings with similar spike amplitudes and burst firings with diminished spike amplitudes from the same ORN. **c**, Representative clusters of spikes recorded from an ORN soma. Waveforms were overlaid at the onset (black traces), which could be further separated into multiple clusters by amplitudes (coloured), although a possibility remains of suctioning axons of adjacent ORNs that have different amplitudes. These collective spikes were all detected and analysed as a whole by thresholding above the noise level with obvious separation of amplitudes. **d,** Representative EOG traces recorded *in vivo* under repetitive odour stimulation. **e,** The relative amplitudes of EOG responses normalized to the first response. n = 3 each for Het and KO mice. **f,g**, Representative traces and power spectra (grey box) of 2-Hz mechanically induced firings in ORNs of Het and KO mice. Het neurons showed rhythmic peaks at the 2^n power. Stim, stimulation; Resp, responses; #, 60-Hz power line hum. **h-k**, Representative traces and time courses of mechanically induced firings in Het (**h,i**) and KO (**j,k**) neurons before (Pre) and after (BPIPP) the application of the AC inhibitor BPIPP. Basal and Post were calculated with 1-min bins, and Stim was calculated by averaging the frequencies over a 20-s stimulation period. One-sided paired T-test before and after BPIPP; P = 0.078, 0.0074 and 0.48 for 3 Het neurons; P = 0.47, 0.0011 and 0.19 for 5 KO neurons. **l**, Spontaneous and mechanically induced firing rates in the presence of another AC inhibitor, SQ 22,536 (SQ). Two-sided unpaired T-test; Basal vs Stim; P = 0.93, n= 14 ORNs from 3 Het mice and P = 0.78, n = 11 ORNs from 3 KO mice. Mean ± s.d. for (**i,k,l**). NS; no significant difference. ** P <0.01.

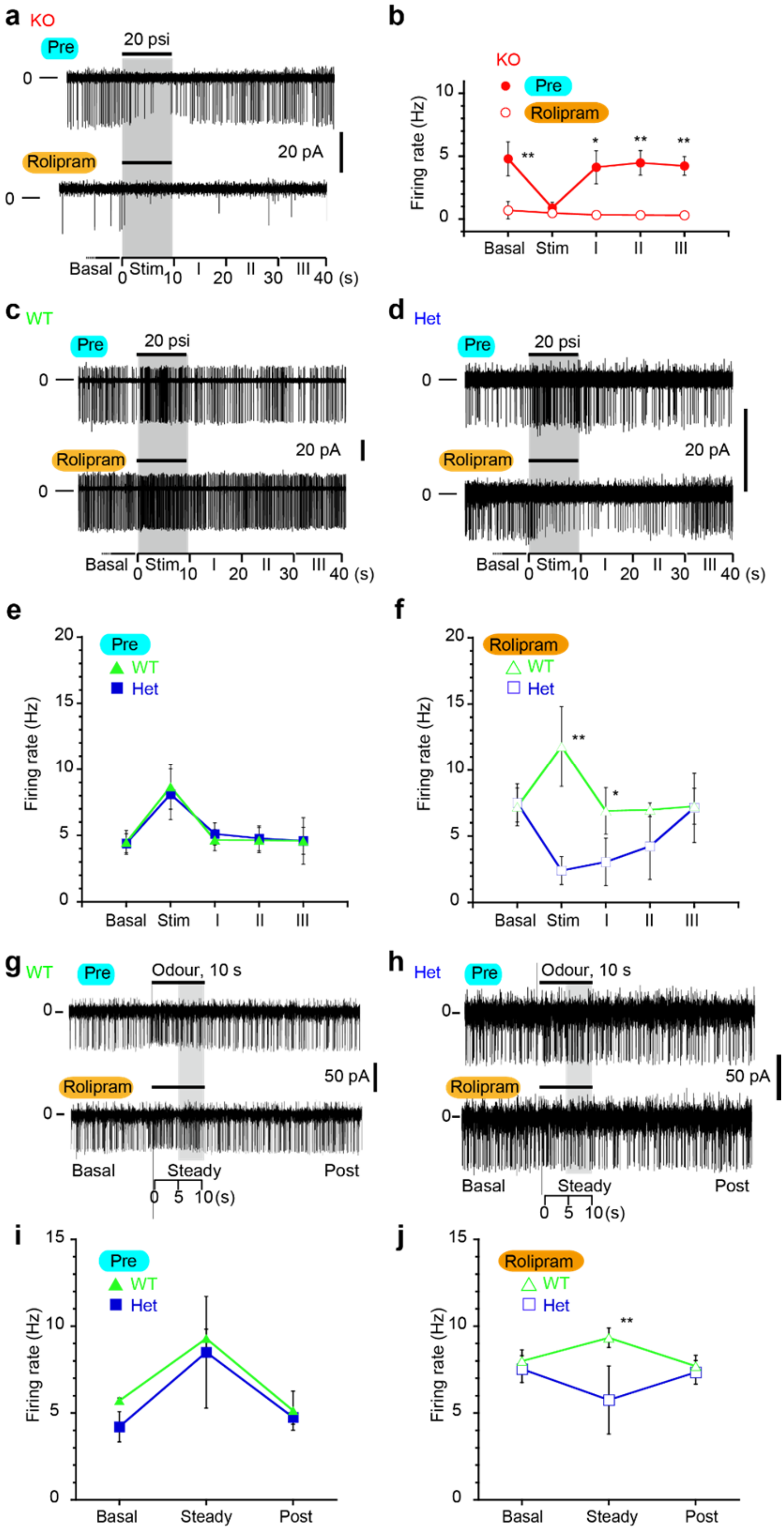

a KO
Pre
20 psi
Rolipram
20 pA
Basal Stim 0 10 I 20 II 30 III 40 (s)
b
KO
Pre
Rolipram
Firing rate (Hz)
Basal Stim I II III
c WT
d Het
e
f
Firing rate (Hz)
WT
Het
g WT
h Het
Odour, 10 s
50 pA
Basal Steady Post
0 5 10(s)
i
j

Supplementary Figure 6

PDEi rolipram attenuates the basal cAMP-buffering capacity.

a,b, Representative traces (**a**) and time course summary (**b**) of the spontaneous- and mechanically induced firing rates of KO neurons in response to puff stimulation (200-ms duration, 2 Hz, 10 s) before (Pre) and after (Rolipram) the application of 10 μM rolipram. The spontaneous firing rates before and after stimulation (Basal) were calculated in 1-min bins, and the parastimulatory firing rates were calculated in 10-s bins (I – III) here and in subsequent panels. One-way paired T-test; P = 0.007529, 0.144532, 0.013562, 0.00462 and 0.001201 for Basal, Stim, and I-III, respectively. n = 4 KO neurons. **c,d,** Representative traces of the spontaneous- and mechanically induced firing rates of WT and Het neurons in response to puff stimulation (200-ms duration, 2 Hz, 10 s) before and after the application of 10 μM rolipram. **e, f,** Summary of the firings of WT and Het neurons before (Pre, **e**) and after (Rolipram, **f**) the application of rolipram in (**c,d**). n = 4 recordings each. **g,h,** Representative traces of the spontaneous- and odourant-induced firing rates of WT and Het neurons (200-ms duration, 2 Hz, 10 s) before and after the application of 10 μM rolipram. **i,j,** Summary of the firings of WT and Het neurons before (Pre, **i**) and after (Rolipram, **j**) the application of rolipram in (**g, h**). n = 5 recordings each, * $P < 0.05$, ** $P < 0.01$. Mean ± s.d. Statistics: **e,f,** Mechanical stimulation; unpaired one-sided T-tests for Basal, Stim, and I-III versus Pre, *P* = 0.458513, 0.341173, 0.22632, 0.426638 and 0.495317, respectively; and versus rolipram, *P* = 0.387744, 0.002638, 0.01101, 0.055813 and 0.47107, respectively. **i,j,** Odourant stimulation; unpaired one-sided T-tests for Basal, Steady, and Post versus Pre, *P* = 0.13609, 0.28401 and 0.280722, respectively; and versus rolipram, *P* = 0.25433, 0.0008 and 0.47374, respectively.

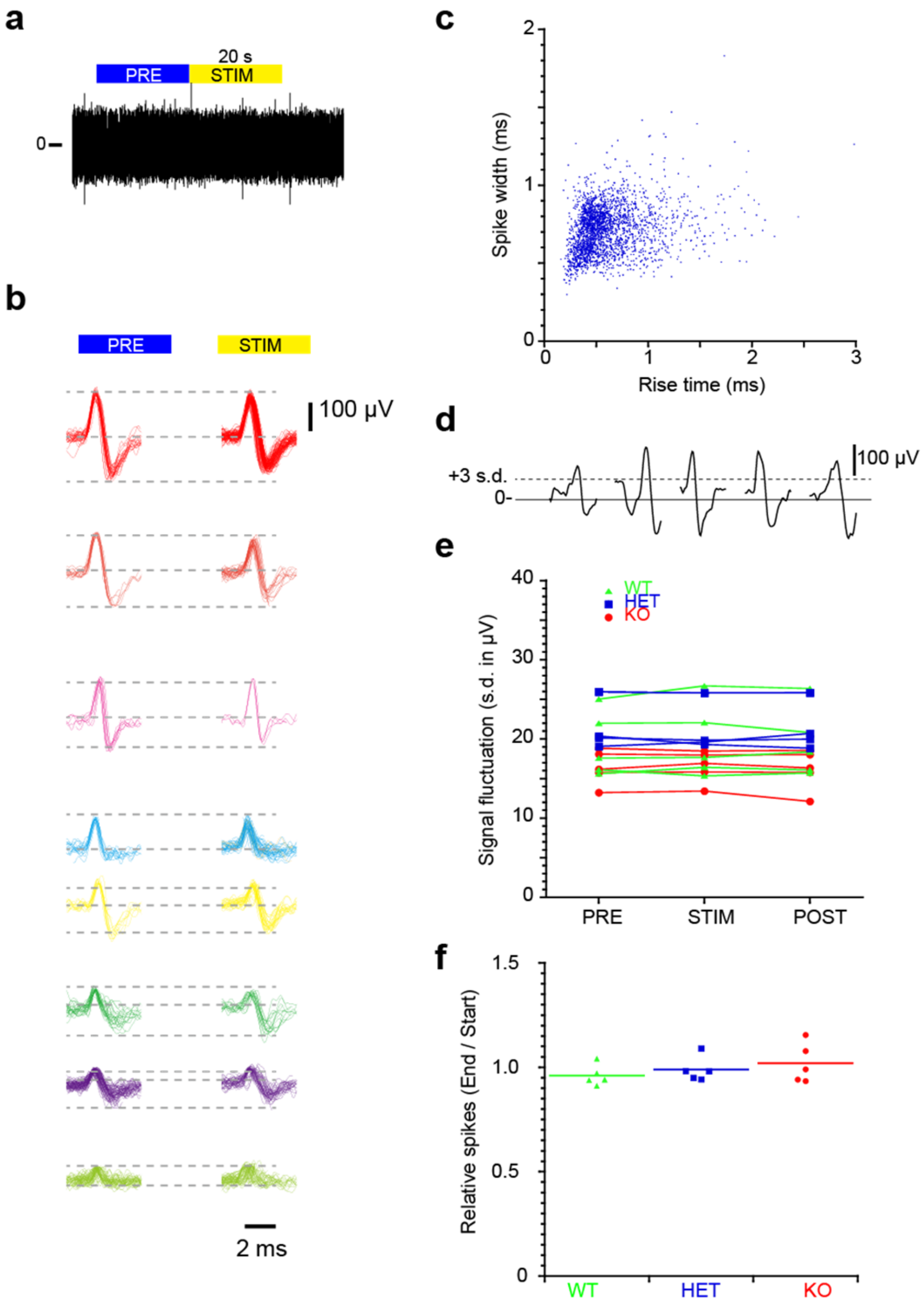

a
20 s
PRE
STIM
0
b
PRE
STIM
100 µV
2 ms
c
Spike width (ms)
Rise time (ms)
d
+3 s.d.
0-
100 µV
e
WT
HET
KO
Signal fluctuation (s.d. in µV)
PRE
STIM
POST
f
Relative spikes (End / Start)
WT
HET
KO

Supplementary Figure 7

Multiunit activities from nerves of ORNs are stably recorded *in vivo*.

a, Representative traces of recorded multiunit activities; the same as those in Fig. 6d. **b**, Spikes can be clustered into several groups by their amplitude, rise time and width, indicating the recording from multiple nerve fibres of ORNs. The shapes of the spikes slightly changed during stimulation. **c,** Distribution of the width and rise times of the spikes in (**a**). No obvious features were found. **d,** Representative traces of abnormally shaped complex action potentials. The collective firing activities were detected by thresholding above +3 s.d. of the basal signal fluctuations defined as the noise level. **e,** The s.d. values of the signals were stable during recording. The values were obtained from the data in the 20-s pre-stimulatory period (PRE), the 20-s stimulatory period (STIM) and the 20-s post-stimulatory period (POST). The s.d. values in PRE were used for thresholding. **f,** Stable collective activities were recorded over 3 min. The activities at the end of the recording (180 - 190 s) were compared to those in the starting period (0 - 10 s). Statistics: (**e**) No significant difference was observed in the basal fluctuations during the recording. *A priori* paired two-sided T-test, $P_{\mathrm{PRE\ vs\ STIM}}$ = 0.42, 0.43 or 0.53; $P_{\mathrm{PRE\ vs\ POST}}$ = 0.68, 0.93 or 0.34; $P_{\mathrm{STIM\ vs\ POST}}$ = 0.63, 0.24 or 0.54 in WT, Het or KO mice, respectively, n = 5 each. (**f**) No significant difference was observed in the stability of recordings among WT, Het or KO mice in baseline stability; one-way ANOVA; $F(3, 12) = 0.885$, $P = 0.43$; *post hoc* Bonferroni multiple comparison, $P_{\mathrm{WT\ vs\ Het}} > 0.999$, $P_{\mathrm{WT\ vs\ KO}} = 0.63$, $P_{\mathrm{Het\ vs\ KO}} > 0.999$. n = 5 each for WT, Het and KO.

Supplementary Note to Fig. 3h-j

The concentrations of each compartment in the membrane (C_1) and OMP domain (C_2) are defined hereafter as $C_1(t)$ and $C_2(t)$ or simply C_1 and C_2, respectively. The rate constants are designated for cytosolic diffusion (k_1), hydrolysis by a phosphodiesterase (PDE: k_2), and binding by OMP (k_3), as shown in Fig. 3h,i. The reverse diffusion of cAMP from the cytosol to C_1 and C_2 to C_1 is ignored. OMP should dissociate cAMP at equilibrium in the long term, which is ignored for simplicity, as the competitive assay results indicated potential hysteresis and that dissociation was slower than association.

Effect of OMP in the membrane compartment

The phasic cAMP surge in the membrane compartment (C_1), such as that triggered by UV uncaging, is shown as follows:

$$\frac{dC_1}{dt} = -k_1 \cdot C_1 - k_2 \cdot C_1 - k_3 \cdot C_1 \qquad (1)$$

The Laplace transform of the equation is

$$s \cdot D_1(t) - D_1(0) = -(k_1 + k_2 + k_3) \cdot D_1(t) \qquad (2)$$

where $D_1(0)$ is the initial concentration of cAMP.
$D_1(\mathrm{t})$ can be solved as follows:

$$D_1(t) = \frac{D_1(0)}{s + k_1 + k_2 + k_3} \qquad (3)$$

Then, considering $D_1(0) = C_1(0)$ in the presence of OMP,

$$C_1(t) = C_1(0) \cdot e^{-(k_1+k_2+k_3)\cdot t} \qquad (4)$$

When OMP is absent, k3 is omitted, and $C_1(t)$ is shown as

$$C_1(t) = C_1(0) \cdot e^{-(k_1+k_2)\cdot t} \qquad (5)$$

Comparing these two equations, the buffering effect of OMP (k3) can accelerate the elimination of cAMP in C_1, leading to a rapid decay of the currents through the membrane-bound CNG2A channels (Fig. 3h-j).
