# Supplementary material for: Olfactory marker protein directly buffers cAMP to avoid depolarization-induced silencing of olfactory receptor neurons
Source: Nat Commun. 2020 May 4;11:2188. doi: 10.1038/s41467-020-15917-2 (PMC7198493; doi:10.1038/s41467-020-15917-2)
Supplement: Supplementary file 3 — Description of Additional Supplementary Files [file 41467_2020_15917_MOESM3_ESM.docx]

**Description for Additional Supplementary Files**

**Supplementary Movie 1.** 3D model of the association between OMP and cAMP. Bound conformational model of OMP and two cAMP molecules rotated around the Z-axis.

**Supplementary Movie 2.** 3D model of OMP compared across vertebrate species. The consensus amino acids of OMP among vertebrates are coloured in pink.

**Supplementary Movie 3.** Mechanical stimulation of ORNs with the solution flow at 20 psi.

**Supplementary Movie 4.** Mechanical stimulation of ORNs with the solution flow at 40 psi.

**Supplementary Movie 5.** Identifying a visible object as food by sniffing prior to PDEi administration in WT mice.

**Supplementary Movie 6.** Identifying a visible object as food prior to PDEi administration in Het mice.

**Supplementary Movie 7.** Identifying a visible object as food by sniffing in WT mice challenged with a PDEi.

**Supplementary Movie 8.** Failure to identify a visible object as food by sniffing in Het mice challenged with PDEi.
